# Food Allergy Prevalence and Characteristics Among Adults in Cyprus: Effects on Health-Related Quality of Life

**DOI:** 10.3390/nu17122028

**Published:** 2025-06-18

**Authors:** Stella A. Nicolaou, Alisa Thuy Anh Pham, Afroditi Alexandrou, Elena Andreou, Elena C. Papanastasiou, Nicolaos Nicolaou

**Affiliations:** 1 Department of Life Sciences, School of Life and Health Sciences, University of Nicosia, 2417 Nicosia, Cyprus; 2 Department of Basic and Clinical Sciences, University of Nicosia Medical School, University of Nicosia, 2408 Nicosia, Cyprus; 3 Department of Education, School of Education, University of Nicosia, 2417 Nicosia, Cyprus

**Keywords:** food allergy, health-related quality of life (HRQL), adults, Cyprus

## Abstract

**Background/Objectives**: Food allergy (FA) is an increasing global concern, yet its prevalence, characteristics, and management vary across populations. Country-specific differences have also been observed in the health-related quality of life (HRQL) of patients with FAs. This study aimed to determine the prevalence and characteristics of FAs among Cypriot adults, aged 18–39 years, and explore its effects on HRQL. **Methods**: A total of 939 randomly selected adults attending universities and colleges across Cyprus completed a questionnaire on demographics and allergy status. Those reporting FA (*n* = 67, 7.1%) completed the Food Allergy Quality of Life Questionnaire-Adult Form (FAQLQ-AF). The results were analyzed using descriptive and inferential statistics. **Results**: Fruits/vegetables (40.5%) and seafood (12.6%) were the most common allergens, and 34.3% of participants reported multiple food allergies. Most participants (94%) experienced symptoms within two hours of allergen exposure, yet only 1.5% carried an epinephrine auto-injector, raising concerns regarding access to allergists or the confirmation of IgE-mediated FA. The mean FAQLQ-AF score was 3.32 ± 0.5 (on a scale of 1–7), indicating mild overall impairment. HRQL impairment was greatest in the Food Allergy-Related Health (FAH) domain and lowest in the Avoidance and Dietary Restrictions (AADR) domain, with participants with multiple allergies, concomitant allergic conditions, or severe symptoms reporting the greatest impacts. **Conclusions**: FA is the most commonly reported allergic disease amongst Cypriot adults and significantly affects their HRQL. The limited confirmation of FA diagnoses through objective methods and the inadequate management of such conditions highlight the need for improved education and access to allergy care for both healthcare providers and patients.

## 1. Introduction

Food allergy (FA) may affect from 6% (physician-diagnosed) to 20% (self-reported) of the general population, and with an increase in its diagnosis in recent years, it is becoming a mounting health problem worldwide [1,2]. In fact, a significant increase in the burden of the disease on national health systems, as well as on patients’ financial circumstances, has been observed [3]. FA presents with a spectrum of symptoms ranging from mild skin or gastrointestinal involvement to severe respiratory or cardiovascular compromise. Diagnosis is not always straightforward, and an oral food challenge (OFC) is often needed to confirm FA. Education of the patient on how to avoid the offending food allergen, to recognize early symptoms of an allergic reaction, and to initiate appropriate treatment remains the mainstay of management [4,5]. Although the majority of reactions to foods are not life-threatening, some cases can be fatal, with serious lasting physical and psychological impacts on affected individuals and their families [4,5].

The prevalence of FA differs between geographical areas, and this has been attributed to differences in dietary habits and interactions with allergens [6]. According to studies on FA in Europe, the prevalence of allergies varies across regions, reflecting dietary habits and differences in pollen sensitization that may drive food allergies. Specifically, allergies to cow’s milk, egg, wheat, soy, peanut, tree nuts, fish, and shellfish appeared to have a higher prevalence in Northern Europe compared to other regions, whereas allergies to peanut and soy were more frequent in Western Europe [7,8]. Data from Southern and Eastern Europe are limited. In Cyprus, the southeastern part of Europe, a few studies conducted mainly in children have indicated that allergies on the island are on the rise, especially in rural areas, pointing out that lifestyle and environmental factors may be significant contributors to this phenomenon [9,10,11,12].

Health-related quality of life (HRQL) is a vital aspect of FA research, as it reflects the broader psychosocial and practical impacts of living with the condition. Understanding these factors can inform patient-centered care strategies and public health interventions. A small number of studies have investigated the HRQL of adults in several countries and have shown that people suffering from FAs show a reduction in their HRQL and an increasing level of anxiety [13,14,15,16,17,18,19,20]. It is also possible for people with the same symptoms to experience different degrees of impairment, while even a self-perceived FA may impair HRQL as much as a confirmed FA [21,22]. Recent research has further emphasized the need for HRQL as a core outcome in FA studies to better capture patient experiences and inform care strategies [23]. Evaluating HRQL has been challenging in the past, and a number of instruments were used; Antolín-Amérigo and colleagues (2016) provide a good review of adult and age-specific questionnaires [19]. Further, as part of the EU project EuroPreval (The Prevalence, Cost and Basis of FA across Europe), a disease-specific questionnaire has been developed for evaluating adults in eight European countries [20]. The results showed that there is variability across the eight European participating countries, indicating that the instrument is indeed sensitive enough to capture differences between different socioeconomic backgrounds [17,19].

Despite global advances in understanding FA prevalence and management, data from Southern and Eastern Europe remain sparse, particularly among adult populations. This study addresses this critical gap by focusing on Cyprus, a region with unique sociocultural and environmental factors that may influence FA trends. Therefore, the current study investigates the prevalence and characteristics of food allergies affecting Cypriot adults, aged 18–39, and their impact on HRQL.

## 2. Materials and Methods

### 2.1. Study Participants and Data Collection

This cross-sectional study was conducted by surveying randomly selected adults aged 18–39 years attending universities and colleges across Cyprus (*n* = 939). This range is consistent with its use in contemporary allergy research [24,25,26]. Participants were asked to complete the study questionnaire (online supplement) to provide information on demographic data, including sex, age, area of residence (urban and rural areas of the 5 main cities in Cyprus: Nicosia, Limassol, Larnaca, Paphos, and Ammochostos), and the presence of allergic disease. In those subjects reporting FA, further information regarding the offending food allergens, the clinical presentation (time of onset from ingestion, type of symptoms, treatment administered), the method of diagnosis (history, skin prick test/serum specific IgE, OFC), and the long-term management plan (avoidance, antihistamine, steroids, adrenaline) was gathered.

### 2.2. The HRQL Questionnaire

To determine the HRQL of adults aged 18–39 years with food allergies, the Food Allergy Quality of Life Questionnaire-Adult Form (FAQLQ-AF) was utilized. This was developed and validated by Flokstra-de Blok and colleagues (2009) and was shown to have longitudinal validity and responsiveness in people following a double-blind, placebo-controlled food challenge [20,22]. The questionnaire was translated and used previously in the English and Greek languages and, as such, was used for the purposes of this study [17]. The questionnaire included 29 questions and used a scale of 1–7, where 1 = no impairment and 7 = maximum impairment. Data were analyzed using the four domains: Domain 1: Avoidance and Dietary Restrictions (AADR); Domain 2: Emotional Impact (EI); Domain 3: Risk of Accidental Exposure (RAE); and Domain 4: FA-related Health (FAH) [20]. Symptoms were classified using a modified Mueller classification as previously reported [27].

Project approval was provided by the Cyprus National Bioethics Committee (ΕΕΒΚ/ΕΠ/2016/01.39). Informed consent was obtained from all study participants.

### 2.3. Statistical Analysis

The targeted sample size was estimated to be satisfactory for determining the associations of interest in a representative sample of the young adult Cypriot population, giving a 95% confidence level with a maximum statistical error margin of ±3.2. In addition to the use of descriptive statistics, inferential statistics were performed by using the Chi-square and, where appropriate, the Fisher exact test and one-way ANOVAs. A *p* value of <0.05 was considered statistically significant. Analyses were performed using SPSS statistics, Version 25.0 (IBM Corp., Armonk, NY, USA). A minimal clinically important difference (MCID) of 0.5 was considered significant, as previously reported for the MCID of HRQL questionnaires with a 7-point scale [28,29].

## 3. Results

### 3.1. Prevalence of Allergic Disease and FA

Of the 939 adults aged 18–39 years who completed the study questionnaire, 16.7% (*n* = 157) reported that they suffer from at least one allergic disease. Males accounted for 46.2% of the sample, while the majority of the participants (54.4%) were 18–22 years old and lived in urban locations (59.7%). A detailed breakdown of the demographics of the study participants is presented in Table 1. Allergies were more common in males than females (Χ^2^(1) = 6.588, *p* = 0.01). Furthermore, the incidence of allergies varied depending on the place of residence, with Nicosia (the capital) showing the greatest percentage of allergic individuals (20.4%, *p* = 0.037). However, no difference in the prevalence of any allergic disease was observed in people living in urban compared to rural locations (Χ^2^(1) = 2.540, *p* = 0.111).

**Figure 1:**
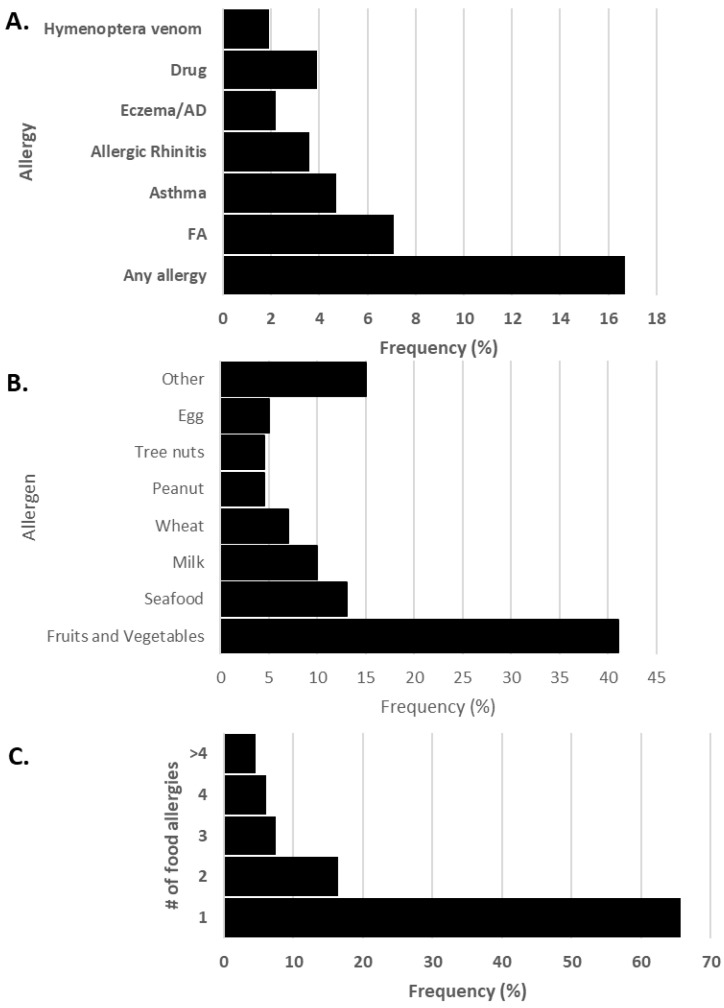
(**A**) Frequency of allergies in adults aged 18–39 years, (**B**) frequency of food allergens and (**C**) number of reported food allergies. AD: atopic dermatitis, FA: food allergy.

FA was the most prevalent allergy reported in both sexes (7.1%), followed by asthma (4.7%) (Figure 1 and Supplementary Table S1). Compared to subjects without food allergies, those with FAs were significantly more commonly affected by other allergic diseases, except for allergic rhinitis (*p* = 0.097; asthma *p* ≤ 0.001; eczema/atopic dermatitis *p* ≤ 0.001; drug allergy *p* = 0.003; and hymenoptera venom allergy *p* = 0.001).

### 3.2. Food Allergy Characteristics, Symptoms, Diagnosis, and Management

The majority of food-allergic individuals reported an allergy to only one food allergen (65.7%), while 12% were allergic to three or more foods. Fruits and vegetables (40.5%) were the most frequently reported allergens, followed by seafood (12.6%) and milk (9.9%) (Figure 1). Peach and kiwi were the most common fruit allergens, and tomato the most prevalent vegetable. Shellfish was the most common in the seafood category (Supplementary Table S2).

Most subjects (94%) developed symptoms within minutes up to 2 h of exposure to the offending food allergen. The most common systems affected were the skin and mucus membranes, with 65.7% and 65.6%, respectively (Supplementary Table S3). More than half of the subjects (59.7%) reported receiving their FA diagnosis from a doctor (Supplementary Table S4). Diagnosis was based on clinical history in 34.3% of the subjects, and in 23.9%, it was confirmed by an OFC (Supplementary Table S3). According to food-allergic patients, the majority of reactions resolved spontaneously (50.7%). For acute management, 34.3% received an antihistamine, 19.4% steroids, and only 6% intramuscular adrenaline. In the long term, the majority of food allergies were managed through avoidance of the offending food allergen (89.6%). Nine (13.4%) subjects carried an antihistamine, one carried steroids, and only one subject carried an adrenaline auto-injector device.

### 3.3. HRQL in Adults with Food Allergies

To measure the HRQL of young people with food allergies, data were collected using the FAQLQ-AF questionnaire [20,30]. On average, the participants in the study indicated that their HRQL was mildly affected (3.32 ± 0.5) by their allergies, with no statistically significant differences between the two sexes (males 3.19 vs. females 3.34). The highest value, and as such, the highest impairment, was in the domain of FAH, and the lowest impairment was in the domain of AADR (Table 2). There were no statistically significant differences between males and females in all categories, indicating a similar level of impairment between the two sexes (Table 2). However, looking at the scores and keeping in mind that a score of 1–3.49 is considered low impairment, 3.50–4.49 medium impairment, and 4.5–7 high impairment, the data show something different. Specifically, females scored higher than 3.5 in 13 out of the 29 questions, whilst males scored higher than 3.5 in 9 out of the 29 questions, predominantly in the AADR and FAH domains (shown in bold in Table 2). For males, hesitating to eat a product, accidentally eating the wrong food, and worrying about health were the most disruptive factors contributing to HRQL impairment. For females, accidentally eating the wrong food, worrying about health, and incomplete labels were the most disruptive factors contributing to HRQL impairment (Table 2).

As mentioned above, the food-allergic individuals were placed in sub-categories based on the level of impairment (Low, Medium, High). Our data indicated that almost 40% of food-allergic individuals experienced either medium or high impairment in all domains (Table 2). Not surprisingly, the highest level of impairment was seen in the FAH domain, with 50% of females stating that their life was impaired (Table 3).

### 3.4. HRQL and Number of Food Allergies and Concomitant Allergic Disorders

To compare HRQL categories, the MCID and statistical significance were considered. As previously reported, the MCID of the HRQL questionnaires with a 7-point scale is approximately 0.5, and this cut-off was used for the interpretation of the current questionnaire used [28,29]. This is an important cut-off point as it determines clinical relevance for the patient and the need for intervention. Although statistical significance was not reached, participants with four or more food allergies showed an increase in all four domains, with the most prominent increase in AADR (4.5 in people with ≥4 food allergies vs. 3.1 in people with ≤3 food allergies). Similar numbers were found in the other domains (Table 3).

**Figure 2:**
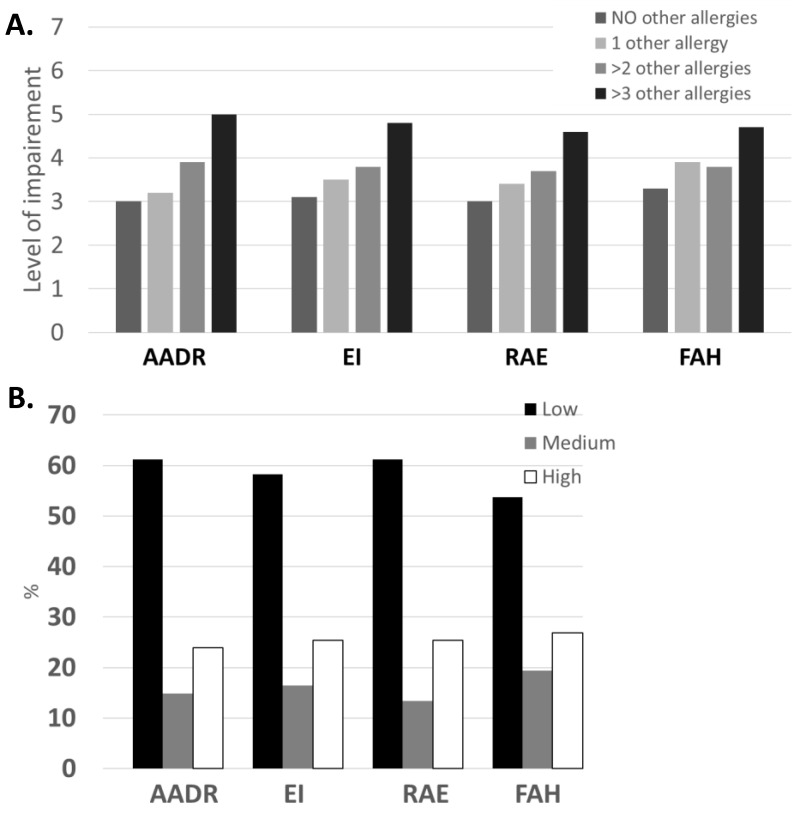
(**A**) Domains of the FAQLQ-AF on a 7-point scale categorized according to the level of impairment (1–3.49 low; 3.5–4.49 medium; 4.5–7.0 high). (**B**) The percentage of people with FA using the domains of the FAQLQ-AF on a 7-point scale categorized according to the level of impairment. AADR: Avoidance and Dietary Restrictions; EI: Emotional Impact; RAE: Risk of Accidental Exposure; FAH: Food Allergy-related Health.

Similarly, examination of the HRQL of people with other allergic disorders showed that as the number of allergic diseases increases, so does the level of impairment. Importantly, once they reach two or more allergies (in addition to their FAs), the impairment increases beyond the 0.5 MCID value, and for people with greater than three allergies, the increase is even more pronounced (Figure 2).

### 3.5. HRQL Severity of Symptoms of Food Allergies and Diagnosis

The reported symptoms were listed in increasing order of impairment of HRQL (lowest to highest). The majority of the participants suffered from skin manifestations and exhibited mucus membrane symptoms (64.7% and 51.5%). Notably, those symptoms did not have the greatest impact on HRQL, although FAH was statistically significant (*p* ≤ 0.043). In fact, people who suffered from cardiovascular as well as respiratory symptoms showed the highest level of impairment, with all domains rated between 4.4 and 5.0, indicating a medium–high level of impairment (Table 4).

Importantly, a higher HRQL was observed in individuals who were diagnosed by a doctor (60%) vs. those who were not diagnosed by a doctor (40%). This was statistically significant in all domains (AADR: 2.3 vs. 3.8 *p* = 0.0001; EI: 2.7 vs. 3.8, *p* = 0.004; RAE: 2.5 vs. 3.7 *p* = 0.003; FAH: 2.9 vs 4.0 *p* = 0.008). Interestingly, the symptoms did not differ between the two groups.

## 4. Discussion

The current study provides the first data on the prevalence and characteristics of FA in the adult Cypriot population and its significant effect on HRQL. Amongst allergic diseases, FA is the most common allergy reported in adults aged 18–39 years in Cyprus (7.1%), with fruits/vegetables (40.5%) and seafood (12.6%) being the most prevalent food allergens. Food-allergic patients have more concomitant allergies compared to subjects from the general young adult population without FA. Almost all subjects (94%) with FA developed symptoms within 2 h of exposure, with skin and mucous symptoms being most common. Although respiratory and cardiovascular symptoms were often reported, the majority of reactions resolved spontaneously. In 23.9% of subjects, FA was confirmed by an OFC, and only one subject carried an adrenaline auto-injector device, indicating that FA remains largely underdiagnosed and undermanaged in the population, as noted in current international guidelines [4,5]. Further, this study reveals that the life of adults aged 18–39 years living with food allergies is impaired, with the highest level of impairment seen in people with multiple food allergies, concomitant allergic disorders, and symptoms associated with the cardiovascular and respiratory systems. This was also observed by Patel and colleagues (2022), who highlighted the compound burden individuals with more severe allergic responses experience [14].

### 4.1. Food Allergy Prevalence

A systematic review and meta-analysis conducted in 2022 on the epidemiology of FA in Europe showed that the overall pooled lifetime and point prevalence of self-reported FA in adults was 22.8% and 12.3%, respectively, with the highest prevalence observed in Eastern Europe [1]. The prevalence observed in Cyprus (7.1%) is similar to that in Greece (6.5%) [1], while in other countries in the region, it varies from 3.2% (in Lebanon) to 9.5% (in Turkey) [31,32]. This reflects the wide variation in the prevalence of FA reported across the region.

Food allergies have been attributed to environmental and lifestyle factors in Europe and in Cypriot allergic children [9,10,11,12]. Age also plays a significant role, with cow’s milk and egg allergies more commonly seen in younger children, whilst among the older age groups, allergies to peanut, tree nuts, fish, and shellfish are more prevalent [33]. Although the data suggest a similar trend to that observed in Greece, some differences exist overall. Specifically, fruits and vegetables were the most common food allergens in Greece (23.5%), compared to 40.5% in Cyprus, followed by seafood (10.7% in Greece, 12.6% in Cyprus) [34]. What is evident from the above comparisons is that although the corresponding percentages in seafood are quite similar in the two countries, the population in Cyprus has almost twice as many people with fruit and vegetable allergies compared to Greece. It remains unclear where these differences stem from, although it was noted that regions with a focus on fruits and vegetables and seafood consumption, such as Cyprus, report higher prevalence rates for these food allergens [35].

Although the future of FA management points to immunotherapy, both the EAACI guidelines and the present study indicate that long-term management relies on avoidance of the offending food allergen [4,35]. Notably, while strict avoidance regimes are effective, they may contribute to widening nutritional deficits and increase anxiety about food consumption [35,36]. In the current study, the prescription of adrenaline was limited, reflecting the considerable variations in prescription practices of adrenaline. Anecdotal accounts suggest that the majority of patients in Cyprus do not receive a proper management plan, and as such, adrenaline use is underrepresented. Interestingly, studies have indicated that the prescription of an adrenaline auto-injector (EAI) indicated worse levels of HRQL [16].

### 4.2. HRQL in Adults with FA

Although the studies on HRQL in food-allergic adults are limited, cross-cultural studies conducted to research HRQL in adults found significant differences between countries [16,17,18,20]. Social determinants, including income, health practices, and healthcare access, contribute to differences in prevalence and management outcomes. In fact, participants in countries with robust healthcare systems, such as Sweden, reported less impairment [37]. Similarly, Santos and colleagues (2023) highlighted the role of accessibility in HRQL, with caregivers and patients from regions with limited infrastructure experiencing greater burdens related to allergen avoidance and dietary restrictions [15]. In contrast to this, the highest levels of impairment were recorded in US adults, who exhibited similar characteristics to Dutch participants [30]. The study also supported the notion that the perception of HRQL may be experienced differently and may be influenced by cultural factors [30]. A study investigating the HRQL of adults in eight European countries found that participants from different countries did not have a comparable HRQL, whilst Sweden had the highest (5.0) and Iceland the lowest impairment (3.2) [17]. Our data place the Cypriot population at the lower end of the spectrum, as the average was 3.32. In regard to MCID, no difference was seen in Iceland, the Netherlands, Poland, France, Spain, and Italy. Although the study suggested that the differences were due to culture and traditions related to eating, Greece, which is considered the most culturally similar to Cyprus, had a higher HRQL (4.0), showing an average increase of 0.7 [17,19]. As such, other socioeconomic factors could be in play here and warrant further investigation. The score in Sweden was 1.7 points higher than that in Cyprus as well. In seven out of eight countries, two of the highest-rated items were under the AADR domain (must personally check whether you can eat something when eating out) and one under the EI domain (fear of an allergic reaction), and we found the same for the Cypriot population [17]. However, we found two additional questions under the FAH domain that they did not.

As previously reported, HRQL is affected by the number of food allergies [14,20]. Similarly, in the present study, participants with four or more food allergies showed an increase in all four domains without reaching statistical significance, as in the Swedish population [16]. This was probably due to the low number of people with four or more allergies (*n* = 6). However, this study has identified clinical significance, as the MCID was 1.4 and well above the 0.5 limit. Comparable findings were observed by Patel and colleagues (2021), where individuals with multiple food allergies (≥2) reported higher impairment across all FAQLQ domains, with FAH and AADR showing the greatest increases for adults with childhood-onset food allergies (COFAs) [14]. A clinically important MCID was also seen in all domains for people with concomitant allergies. Similar trends were highlighted by Dierick and colleagues (2020), who found that HRQL significantly deteriorates as the number of food and other allergic disorders increases, often leading to disproportional socioeconomic burdens [35]. Other studies have demonstrated that HRQL impairment becomes statistically significant when patients have at least four additional allergies. However, in the present study, this scenario was observed in only one individual, preventing statistical analysis. Likewise, examination of the HRQL of people with other allergic disorders showed that as the number of allergic diseases increases, so does the level of impairment. Importantly, once they have two or more allergies (in addition to their FA), the impairment increases beyond the 0.5 MCID value, and for people with greater than three allergies, the increase is much more pronounced. Indeed, heightened psychosocial burden and emotional impact were observed for individuals managing multiple allergic conditions [13,15]. Protudjer and colleagues (2025) further emphasized that socioeconomic disparities exacerbate these effects, reducing access to allergen-free foods and interventions essential to minimizing HRQL impairments in multi-allergic individuals [37].

Studies have shown that a great portion of food-allergic patients with a severe or mild reaction do not visit a doctor, while physician-confirmed food allergies were associated with lower psychosocial burdens compared to self-reported cases [13,27]. Similarly, we found that the HRQL of people who were diagnosed by a doctor was significantly lower than those who were not. This may be largely attributed to greater education and management efficacy provided by healthcare professionals [13]. Alotiby (2024) further underscored the value of routine counseling in improving preparedness and thus reducing emotional stress in adults with food allergies [38]. Still, HRQL may be as impaired in people with a perceived FA at least as much as in those with a diagnosed allergy [22]. This was also clinically significant, as demonstrated by the MCID value. In our case all four domains reached statistical significance, while in the other study, two out of the four domains reached significance (AADR and EI). Similarly, the symptoms did not differ between the two groups, and it is therefore not surprising that the FAQLQ-AF questionnaire score remained unchanged when given two years later to the same patients [27].

As expected, food-allergic individuals in this study indicated that they experienced a higher level of impairment if the allergy manifested with either respiratory or cardiovascular symptoms but not skin problems (the most commonly observed manifestation), which was perceived as low–medium impairment, with the exception of the FAH domain. Comparable findings were reported in the literature, where the highest HRQL disruption was observed for severe reactions such as wheezing or respiratory distress, reinforcing the greater emotional toll such symptoms impose [15]. Similarly, Patel and colleagues (2021) documented that those with multi-system reactions reported significantly worse HRQL, with FAH consistently being the most impaired domain [14].

In the present study, male and female participants had comparable HRQL, as in the Swedish study, while other studies found sex differences, with females showing slightly greater social burdens and dietary restrictions [13,16,18]. We did, however, find that females developed symptoms faster. Our domain analysis indicated that in the Cypriot population, domain FAH showed the highest impairment, whilst AADR showed the lowest. This was exactly reversed in the Swedish population, in keeping with the differences observed in different countries [16].

The current study has several limitations. One limitation is recall bias, that is, reporting inaccuracies as a result of an extended time lapse between the survey and the allergic episode in some cases. Although the data collected reflect self-perceived food allergies, it has been previously shown that HRQL may be as impaired in people with a perceived FA at least as much as in those with a diagnosed allergy [22]. Another limitation is that the study exclusively recruited participants from universities and colleges, which may limit the representativeness of the study population, as not all adults aged 18–39 years in Cyprus attend higher education. The sample size of our study was also limited in comparison to previous studies in other parts of the world. However, to the best of our knowledge, to date, this is the first study that investigated the prevalence of IgE-mediated food allergies in adults aged 18–39 years from Cyprus due to the scarcity of data currently available in the literature.

## 5. Conclusions

As the prevalence of FA rises, its impact on the lives of affected individuals and their families becomes increasingly evident. This study confirms that FA is the most common allergy reported in adults aged 18–39 years in Cyprus, significantly impairing quality of life, particularly among those with multiple food allergies, concomitant allergic conditions, or severe cardio-respiratory symptoms. Fruits/vegetables and seafood were identified as the most prevalent food allergens. Despite these findings, FA in Cyprus remains largely underdiagnosed and undermanaged, highlighting significant gaps in care.

To address the growing burden of FA, it is critical to implement educational programs for healthcare professionals to enhance diagnostic accuracy and promote adherence to management guidelines. Simultaneously, public awareness campaigns can empower individuals to recognize and manage FA more effectively. Collaborative, multi-sector efforts at the national level, including improved access to diagnostic tools, standardized use of adrenaline auto-injectors, and better allergen labeling practices, are needed to enhance patient care. Future research should further explore cultural, dietary, and healthcare system factors that influence FA prevalence and outcomes. By addressing these gaps and challenges, we can take meaningful steps toward improving clinical care and the quality of life for individuals living with food allergies.

## Figures and Tables

**Table 1:** Demographic characteristics of study population.

	All Participants Participant, % (Number)	Allergic Individuals Participant, % (Number)	Food Allergy Participant, % (Number)
**Total**	100 (939)	16.7 (157)	7.1 (67)
Sex			
Male	46.2 (434)	36.9 (58)	38.8 (26)
Female	53.8 (505)	63.1 (99)	61.2 (41)
**Age group (years)**			
18–22	54.4 (511)	51.6 (81)	50.7 (34)
23–27	34.5 (324)	35.7 (56)	40.3 (27)
28–32	8.4 (79)	8.3 (13)	7.5 (5)
33–39	2.7 (25)	4.5 (7)	1.5 (1)
District of residence *			
Nicosia	51.1 (480)	62.4 (98)	61.2 (41)
Limassol	17.9 (168)	12.7 (20)	14.9 (10)
Larnaca	24.3 (228)	19.7 (31)	17.9 (12)
Paphos	3.7 (35)	2.5 (4)	1.5 (1)
Ammochostos	3.0 (28)	2.5 (4)	4.5 (3)
**Region of Residence**			
Urban	59.7 (561)	54.1 (85)	53.7 (36)
Rural	40.3 (378)	45.9 (72)	46.3 (31)

* Districts of residence include Nicosia, the capital and largest city; Limassol, a southern coastal city; Larnaca, a southeastern coastal city; Paphos, a southwestern coastal city; and Ammochostos, a southeastern region.

**Table 2:** Outcomes of FAQLQ-AF domains in all food-allergic adults aged 18–39 years and categorized by sex.

	Total Mean * (n)	Male Mean (n)	Female Mean (n)
**Domain 1: Allergen Avoidance and Dietary Restriction**			
**Average Score**	3.31	3.14	3.26
Must always be alert as to what you are eating?	**3.51 (67)**	3.44 (25)	** 3.55 (42) **
Are able to eat fewer products?	3.21 (66)	2.68 (25)	** 3.54 (41) ^m^**
Are limited as to the products you can buy?	3.48 (67)	3.12 (25)	** 3.69 (42) ^m^**
Must read labels?	3.45 (67)	3.04 (25)	** 3.69 (42) ^m^**
Must refuse many things during social activities?	2.42 (67)	2.64 (25)	2.29 (42)
Are less able to spontaneously accept an invitation to stay for a meal?	2.56 (66)	2.21 (25)	2.76 (42) **^m^**
Are less able to taste or try various products when eating out?	3.30 (66)	** 3.67 (25) ^m^**	3.10 (42)
Can eat out less?	2.76 (67)	3.20 (25) **^m^**	2.50 (42)
Must personally check whether you can eat something when eating out?	3.37 (67)	3.44 (25)	3.33 (42)
Hesitate eating a product when you have doubts about it?	**3.99 (67)**	** 4.20 (25) **	** 3.86 (42) **
That you must explain to those around you that you have a FA?	3.33 (67)	2.92 (25)	** 3.57 (42) ^m^**
**Percentage of questions ≥ 3.5**	**18%**	** 18% **	** 55% **
**Domain 2: Emotional Impact**			
**Average Score**	3.33	3.24	3.38
Have the feeling that you have less control of what you eat when eating out?	3.30 (67)	3.00 (25)	3.48 (42)
Of an allergic reaction?	**3.81 (67)**	** 3.72 (25) **	** 3.86 (42) **
Of accidentally eating the wrong food?	**4.00 (67)**	** 3.96 (25) **	** 4.02 (42) **
Of an allergic reaction eating our despite the fact that your dietary restrictions?	**3.70 (67)**	** 3.76 (25) **	** 3.67 (42) **
To what degree do you feel you are being a nuisance because you have a FA when eating out?	2.84 (67)	2.92 (25)	2.79 (42)
How discouraged do you feel during an allergic reaction?	2.88 (67)	2.96 (25)	2.83 (42)
How apprehensive are you about eating something you have never eaten before?	2.76 (67)	2.36 (25)	3.00 (42)
**Percentage of questions ≥ 3.5**	**43%**	**43%**	**43%**
**Domain 3: Risk of Accidental Exposure**			
**Average Score**	3.2275	3.15	3.2725
Sometimes frustrate people when they are making an effort to accommodate your FA?	2.46 (67)	2.52 (25)	2.43 (42)
That the ingredients of the product change?	3.07 (67)	3.08 (25)	3.07 (42)
That label is incomplete?	**3.82 (67)**	** 3.72 (25) **	** 3.88 (42) **
That the lettering on the labels is too small?	3.12 (67)	** 3.52 (25) ^m^**	2.88 (42)
That the label states: “May contain (traces of)…”	**3.57 (67)**	3.12 (25)	** 3.83 (42) ^m^**
Those ingredients are different in other countries (for example during vacations)?	3.24 (67)	2.88 (25)	3.45 (42)
That people underestimate your problems caused by FA?	**3.51 (67)**	3.64 (25)	3.43 (42)
For your host or hostess should you have an allergic reaction?	3.03 (67)	2.72 (25)	3.21 (42)
**Percentage of questions ≥ 3.5**	**38%**	** 29% **	** 29% **
**Domain 4: FA and related Health**			
**Average Score**	3.62	3.38333	3.75333
That it is unclear to which foods you are allergic?	3.24 (67)	2.92 (25)	3.43 (42) **^m^**
About your health?	**4.05 (65)**	** 3.91 (25) **	** 4.12 (42) **
That the allergic reactions to foods will become increasingly severe?	**3.57 (67)**	3.32 (25)	** 3.71 (42) **
**Percentage of questions ≥ 3.5**	**67%**	** 33% **	** 67% **

**Bold**—≥3.5 indicating medium/high risk; **^m^**—indicating ≥ 0.5 MCID between males and females. The higher value is labeled. * Scale: 1–7; 1 = no impairment, 7 = maximum impairment.

**Table 3:** Impairment of HRQL using domains of the FAQLQ-AF. The data are categorized according to the level of impairment (1–3.49, low; 3.5–4.49, medium; 4.5–7.0 high) and are expressed as the percentage of people affected (n).

	Total % (n)	Male % (n)	Female % (n)
**Domain 1: Allergen Avoidance and Dietary Restriction**			
Low impairment (1–3.49)	61.2 (41)	64.0 (16)	59.5 (25)
Medium impairment (3.5–4.49)	14. 9 (10)	16.0 (4)	14.3 (6)
High impairment (4.5–7.0)	23.9 (16)	20.0 (5)	16.2 (11)
**Domain 2: Emotional Impact**			
- Low impairment (1–3.49)	58.2 (39)	64.0 (16)	54.8 (23)
- Medium impairment (3.5–4.49)	16.4 (11)	12.0 (3)	19.0 (8)
- High impairment (4.5–7.0)	25.4 (17)	24 (6)	16.2 (11)
**Domain 3: Risk of Accidental Exposure (n)**			
Low impairment (1–3.49)	61.2 (41)	64.0 (16)	59.5 (25)
Medium impairment (3.5–4.49)	13.4 (9)	8.0 (2)	16.7 (7)
High impairment (4.5–7.0)	25.4 (17)	28 (7)	23.8 (10)
**Domain 4: Health-related (n)**			
Low impairment (1–3.49)	53.7 (36)	60.0 (15)	50.0 (21)
Medium impairment (3.5–4.49)	19.4 (13)	16.0 (4)	21.4 (9)
High impairment (4.5–7.0)	26.9 (18)	24.0 (6)	28.6 (12)

**Table 4:** Impairment of HRQL using the domains of the FAQLQ-AF and the number of food allergies and other concomitant allergies. The prevalence as well as the average value is reported for each domain.

	Prevalence % (n)	AADR	EI	RAE	FAH
**Number of food allergies**					
HRQL in people with three or fewer food allergies	91.0 (61)	3.1	3.2	3.1	3.5
HRQL in people with four or more food allergies	9.0 (6)	4.5	4.4	4.2	4.3
**Number of other allergies**					
HRQL of people with no other allergies (*n* = 34)	50.7 (34)	3.0	3.1	3.0	3.3
HRQL of people with 1 other allergy (*n* = 21)	31.3 (21)	3.2	3.5	3.4	3.9
HRQL of people with ≥2 other allergies (*n* = 11)	16.4 (11)	3.9	3.8	3.7	3.8
HRQL of people with ≥3 other allergies (*n* = 5)	7.4 (5)	5.0	4.8	4.6	4.7
**Symptoms—Severity Modified Mueller (0–4)**					
Mucus Membrane (1)	51.5 (35)	3.3	3.2	3.2	3.3
Skin (1)	64.7 (44)	3.5	3.4	3.3	3.9 *
OAS (0)	22.3 (15)	3.6	3.7	3.7	3.9
GI (2)	19.4 (13)	3.8	4.0	3.8	4.0
Respiratory (3)	28.4 (19)	4.4 *	4.4 *	4.5 *	4.4 *
Cardio (4)	11.9 (8)	4.6 *	4.5 *	5.0 *	4.9 *
Diagnosed By a Doctor *					
Yes	60.0 (40)	3.8	3.8	3.7	4.0
No	40.0 (27)	2.3	2.7	2.5	2.9

* *p* < 0.05, Classification of the most severe reaction using a modified Mueller classification: 0 = oral allergy symptoms; 1 = skin/mucous membrane symptoms; 2 = gastrointestinal symptoms; 3 = respiratory symptoms; 4 = cardiovascular symptoms; AADR: Avoidance and Dietary Restrictions; EI: Emotional Impact; RAE: Risk of Accidental Exposure; FAH: Food Allergy-related Health.

## Data Availability

The original contributions presented in this study are included in the article/Supplementary Materials. Further inquiries can be directed to the corresponding author.

## Supplementary Materials

The following supporting information can be downloaded at https://www.mdpi.com/article/10.3390/nu17122028/s1: Table S1: Prevalence of allergic disease in Cypriot adults; Table S2: Frequency of food allergens; Table S3: Classification of symptoms in the FAQLQ-AF; and Table S4: Diagnosis and management of food allergy in affected individuals.

## Abbreviations

The following abbreviations are used in this manuscript:

AADR	Avoidance and Dietary Restrictions
AD	Atopic Dermatitis
EI	Emotional Impact
FA	Food Allergy
FAH	FA related Health
FAQLQ-AF	Food Allergy Quality of Life Questionnaire-Adult Form
HRQL	Health-Related Quality of Life
MCID	Minimal Clinically Important Difference
OFC	Oral Food Challenge
RAE	Risk of Accidental Exposure
