# Supplementary material for: Food Allergy Prevalence and Characteristics Among Adults in Cyprus: Effects on Health-Related Quality of Life
Source: Nutrients. 2025 Jun 18;17(12):2028. doi: 10.3390/nu17122028 (PMC12195849; doi:10.3390/nu17122028)
Supplement: Supplementary file 1 [file nutrients-17-02028-s001.zip › nutrients-3680290-supplementary.pdf]

Supplementary Information

Table S1: Prevalence of Allergic Disease in Cypriot adults

	Participant % (n)	Male % (n)	Female % (n)
Any allergy	16.7 (157)	13.4 (58)	19.6 (99)*
Food Allergy	7.1 (67)	6.0 (26)	8.1 (41)
Asthma	4.7 (44)	3.9 (17)	5.3 (27)
Allergic Rhinitis	3.6 (34)	4.4 (19)	3.0 (15)
Eczema/Atopic Dermatitis	2.2 (21)	1.4 (6)	3.0 (15)
Drug Allergy	3.9 (37)	2.3 (10)	5.3 (27)
Hymenoptera venom allergy (Bee, Wasp)	1.9 (18)	3.0 (13)	1 (5)

*X^2 (1) = 6.588, p = .01

Table S2: Frequency of food allergens

Food Allergen		% (n)	Total %
Milk		9.9 (11)	
Wheat		7.2 (8)	
Egg		5.4 (6)	
Seafood	Shellfish	6.3 (7)	12.6
	Mollusks	4.5 (5)	
	Fish	1.8 (2)	
Peanut		4.5 (5)	
Tree Nuts	Hazelnut	2.7 (3)	4.5
	Pistachio	0.9 (1)	
	Almond	0.9 (1)	
Seeds	Soya	0.9 (1)	3.6
	Pumpkin seed	0.9 (1)	
	Cacao	0.9 (1)	
	Mustard	0.9 (1)	
Fruits and Vegetables	Tomato	8.1 (9)	40.5
	Kiwi	6.3 (7)	
	Peach	6.3 (7)	
	Strawberry	3.6 (4)	
	Banana	2.7 (3)	
	Pineapple	2.7 (3)	
	Citrus fruit	0.9 (1)	
	Olives	0.9 (1)	
	Pear	0.9 (1)	
	Mango	0.9 (1)	
	Avocado	0.9 (1)	
	Mushroom	3.6 (4)	
	Eggplant	1.8 (2)	
	Spinach	0.9 (1)	
Spices	Pepper	2.7 (3)	5.4
	Cinnamon	1.8 (2)	
	Chili	0.9 (1)	
Meat	Pork	1.8 (2)	3.6
	Rabbit	0.9 (1)	
	Lamb	0.9 (1)	
Others	Chocolate	0.9 (1)	2.7
	Honey	0.9 (1)	
	Mint	0.9 (1)	
Number of food allergies	1	65.7 (44)	
	2	16.4 (11)	
	3	7.5 (5)	
	4	6.0 (4)	
	>4	4.5 (3)	

Table S3: Classification of Symptoms in the FAQLQ-AF

	% (n)
**Time of onset**	
<5min	34.4 (23)
Within 30 min	49.3 (33)
Up to 2 hours	10.4 (7)
>2hrs	6.0 (4)
**Specific symptoms/signs**	
Skin	
Itchy skin, rash or hives	65.7 (44)
Oral allergy syndrome (OAS)	
Itching or tingling in the oral cavity	22.4 (15)
Mucous membrane	
Swelling of face, lips or tongue	35.8 (24)
Runny or blocked nose, sneezing	13.4 (9)
Respiratory	
Difficulty in breathing, wheezing or chest tightness	28.4 (19)
Difficulty in swallowing, tightness in throat or hoarse voice	16.4 (11)
Gastrointestinal	
Abdominal pain, vomiting or diarrhea	20.8 (14)
Cardiovascular	
Feeling dizzy or lightheaded	11.9 (8)
	5.9 (4)
Others	

FAQLQ-AF: Food Allergy Quality of Life Questionnaire-Adult Form.

Table S4: Diagnosis and Management of Food Allergy in Affected Individuals

	Participant % (n)
**Diagnosis of FA confirmed by a doctor**	
Yes	59.7 (40)
No	40.3 (27)
**Diagnosis of FA based on History/Allergy testing**	
Only History	34.3 (23)
Skin tests	19.4 (13)
Blood tests	25.4 (17)
Oral Food Challenge	23.9 (16)
**Acute treatment for allergic reactions to foods**	
None	50.7 (34)
Oral Antihistamine	34.3 (23)
Steroids Oral/IV	19.4 (13)
Intramuscular Adrenaline	6.0 (4)
**Long term management plan of FA in FA-subjects**	
Avoidance of the offending food allergen	89.6 (60)
Oral Antihistamine	13.4 (9)
Oral Steroids	1.5 (1)
Adrenaline Auto-Injector	1.5 (1)
None	10.4 (7)
